# Characterization of Retinal VIP-Amacrine Cell Development During the Critical Period

**DOI:** 10.1007/s10571-024-01452-x

**Published:** 2024-02-05

**Authors:** Xuhong Zhang, Xiaoyu Wang, Yanqing Li, Yingying Zhang, Hong Zhu, Chen Xie, Yudong Zhou, Ye Shen, Jianping Tong

**Affiliations:** 1 Department of Ophthalmology, The First Affiliated Hospital of Medical College, Zhejiang University, No.79 Qingchun Road, Shangcheng District, Hangzhou, 310003 Zhejiang China; 2 Center for Brain Research and Brain-Machine Integration, School of Brain Science and Brain Medicine, Zhejiang University, No.866 Yuhangtang Road, Xihu District, Hangzhou, 310058 Zhejiang China

**Keywords:** VIP-ACs, Structure, Development, Electrophysiological properties

## Abstract

Retinal vasoactive intestinal peptide amacrine cells (VIP-ACs) play an important role in various retinal light-mediated pathological processes related to different developmental ocular diseases and even mental disorders. It is important to characterize the developmental changes in VIP-ACs to further elucidate their mechanisms of circuit function. We bred VIP-Cre mice with Ai14 and Ai32 to specifically label retinal VIP-ACs. The VIP-AC soma and spine density generally increased, from postnatal day (P)0 to P35, reaching adult levels at P14 and P28, respectively. The VIP-AC soma density curve was different with the VIP-AC spine density curve. The total retinal VIP content reached a high level plateau at P14 but was decreased in adults. From P14 to P16, the resting membrane potential (RMP) became more negative, and the input resistance decreased. Cell membrane capacitance (MC) showed three peaks at P7, P12 and P16. The RMP and MC reached a stable level similar to the adult level at P18, whereas input resistance reached a stable level at P21. The percentage of sustained voltage-dependent potassium currents peaked at P16 and remained stable thereafter. The spontaneous excitatory postsynaptic current and spontaneous inhibitory postsynaptic current frequencies and amplitudes, as well as charge transfer, peaked at P12 to P16; however, there were also secondary peaks at different time points. In conclusion, we found that the second, third and fourth weeks after birth were important periods of VIP-AC development. Many developmental changes occurred around eye opening. The development of soma, dendrite and electrophysiological properties showed uneven dynamics of progression. Cell differentiation may contribute to soma development whereas the changes of different ion channels may play important role for spine development.

**Graphical Abstract:**

The second, third and fourth weeks after birth were important periods of VIP-AC development. VIP::Ai14 and VIP::Ai32 mice were used for soma and spine analysis, respectively. The developmental curves for VIP-AC soma have a distinct and longer platform, whereas the developmental curves for spine have a longer and smoother slopes. When the number of VIP-AC some is increasing, cell differentiation may play an important role. During the development of spine, the development of different ion channels is the most vital events. Kv-Ka represents the ion channels that conduct Ka, Kv-Kdr represents the ion channels that conduct Kdr, GABAR represents the inhibitory transmission and NMDAR represents the excitatory transmission. The events occur chronologically from left to right.

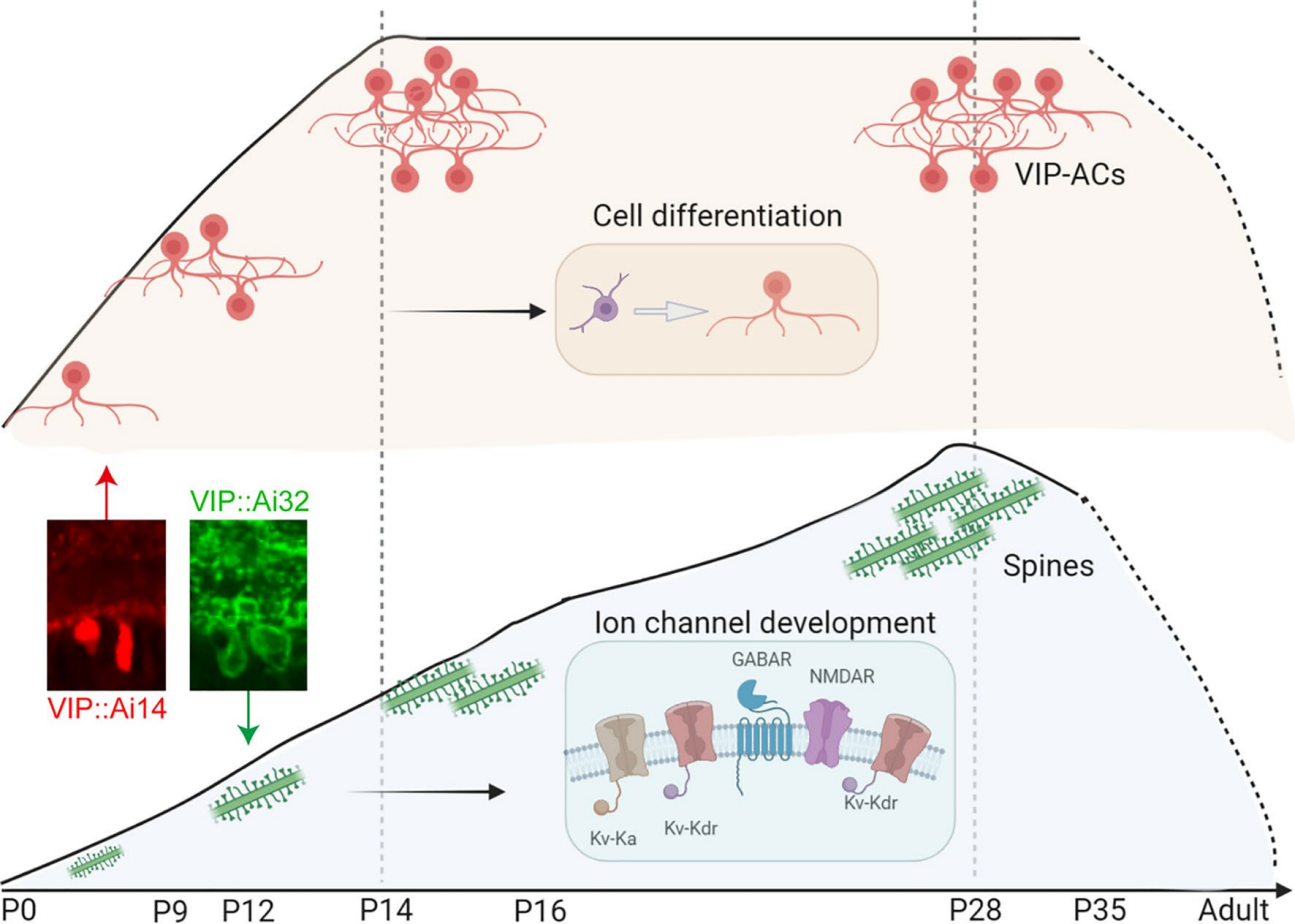

## Introduction

Vasoactive intestinal peptide amacrine cells (VIP-ACs), as one kind of ACs in the retina, exist in a wide range of species (Fukuda et al. 1981a, b; Pachter and Lam 1986; STONE et al. 1988; Li and Lam 1990; Mao and Liu 2013). They were first found in bird retinas in 1979 (Brecha et al. 1979). VIP-ACs have been found to play important roles in many ocular diseases, such as myopia (Mao and Liu 2013), diabetic retinopathy (Giunta et al. 2012), and neuroblastoma (Maugeri et al. 2018). Many of these ocular diseases are related to cell growth and development. These diseases are related to the neuromodulatory function of VIP-ACs (Scuderi et al. 2013) or their inhibitory connections (Ramsey et al. 2007). To better understand the mechanism underlying the role of VIP-ACs in ocular diseases, researchers have explored the structure, subtypes and functions of VIP-ACs with many methods (Akrouh and Kerschensteiner 2015; Park et al. 2015; Perez de Sevilla Muller et al. 2019). They found retinal VIP-ACs have at least three subtypes, as VIP1-3. VIP1 has two layers of dendrites in the inner plexiform layer (IPL) and the soma locates in inner nuclear layer (INL). The soma of VIP2 is also in INL, however, with single of dendrite in IPL. The VIP3 is also monolayer dendrite, but the soma is in the ganglion cell layer. VIP-ACs have connection with bipolar cells, retinal ganglion cells and other ACs. There are also gap junctions among VIP-ACs. All the above observation is conducted at adult stage and the developmental characters is lack. Analysis of mRNA expression has revealed that the VIP content in the retina reaches a peak at approximately the time of eye opening (Bagnoli et al. 2003) and decreases in adults. However, the detailed changes that occur during VIP-AC development in the peri-eye-opening period, especially the changes in the neuron-like electrical properties of VIP-ACs, are still unknown. During the first month of mouse development, growth occurs the fastest. The peri-eye-opening period in mice corresponds to the period of childhood to adolescence in humans (Flurkey and Harrison 2007; Dutta and Sengupta 2016). Therefore, it is necessary to characterize VIP-AC development during the extended peri-eye opening period in detail. We propose that in addition to changes in VIP mRNA content being highly time dependent, the electrophysiological properties of VIP-ACs may also exhibit a specific developmental trend, and we performed detailed assessments of both the density and electrophysiological properties of VIP-ACs during the extended peri-eye-opening period.

## Materials and Methods

### Animals and Procedure

VIP-Cre mice (Zhang et al. 2021a) were bred with Ai32 mice (Madisen et al. 2012) to generate VIP-Cre^+/+^::Ai32^R26EYFP/R26EYFP^ or VIP-Cre^+/−^::Ai32^R26EYFP/WT^ mice (VIP-YFP mice), and then VIP-Cre^+/+^::Ai32^R26EYFP/R26EYFP^mice were bred with Ai14 (Park et al. 2015) mice to produce VIP-Cre^+/−^::Ai32^R26EYFP/WT^::Ai14^R26tdTom/WT^ mice (VIP-tdTomato-YFP mice). VIP-Cre mice were also bred with Ai14 mice to generate VIP-Cre^+/−^::Ai14^R26tdTom/WT^ mice (VIP-tdTomato mice). VIP-tdTomato mice were used for cell morphology observation and electrophysiological analysis, whereas VIP-tdTomato-YFP mice and VIP-YFP mice were used for cell morphology observation and immunofluorescence staining. The mice were housed under standard housing conditions and provided food and water ad libitum. We checked the pregnant mice every day at 10 am. If there were newborn pups and their skin was bright red, they were considered postnatal day (P) 0 pups; if their skin was light red, they were considered P1 pups. We collected samples from the mice at 10 am on P0, P2, P4, P6, P8, P10, P12, P14, P16, P18, P21, P28 and P35 for the experiment. The animal use and care protocols were performed according to the guidelines of Zhejiang University. Use of the laboratory animals was approved by the Tab of Animal Experimental Ethical Inspection of the First Affiliated Hospital, College of Medicine, Zhejiang University (approval No. 2,021,001).

### Intravitreal Virus Injection into Mouse Pups

Time is required for a virus to be expressed after transduction. To ensure that the virus was expressed before observation, it was injected intravitreally into mice on P4. A thin needle (tip diameter of approximately 2–3 μm) drawn with a glass electrode (Borosilicate glass, O.D.: 1.5 mm, I.D.: 0.86 mm, Sutter Instrument) was attached to a 10 mL syringe for injection. VIP-IRES-Cre mouse pups were anesthetized by hypothermia by placing the pups on ice for approximately 30 s, and then 0.5 µL of AAV2/2-hEF1a-DIO-EGFP-WPRE-pA virus was injected into the vitreous cavity to label retinal VIP-ACs. The needle was inserted into the edge of the black part of the eye, indicating the ora serrata. After the needle penetrated the skin, another breakthrough was felt when the needle entered the eyeball. At this time, the liquid level in the needle tip increased, indicating partial liquid outflow from the eye. The virus was slowly and steadily injected, and the needle was kept in place for 20 s to allow the virus to spread before being withdrawn.

### Analysis of Retinal VIP Content by ELISA

After anesthesia, mouse retinas were collected on ice as soon as possible and stored at − 80 °C. The retinas were immersed in 200 µL RIPA lysis buffer (PC101, EpiZyme, Shanghai, China) and ultrasonicated to obtain homogenates. The samples were centrifuged at 4 °C and 2000×*g* for 20 min, and the supernatant was collected. For analysis, samples were added to wells according to the instructions of the ELISA kit manufacturer. Then, 100 µL horseradish peroxidase (HRP)-labeled antibody was added to each well except the blank wells, and the plate was sealed with a sealing membrane and incubated for 60 min in a 37 °C incubator. After the liquid was discarded, the wells were washed with washing solution (350 µL per well) and the plate was allowed to rest for one minute; this process was repeated for a total of 5 times. Substrate A and substrate B (50 µL) were added to each well, and the plate was incubated at 37 °C for 15 min away from light. Finally, 50 µL of stop solution was added to each well, and the OD value of each well was measured at 450 nm within 15 min.

### Western Blotting

Western blotting was performed according to our previously reported protocols (Zhang et al. 2022). In brief, retinas were homogenized in RIPA buffer containing complete protease inhibitor cocktail (Roche). The protein concentration of each sample was determined by a BCA protein assay (Bio-Rad Laboratories). The total protein samples were electrophoresed on 2–20% SDS-PAGE gels and transferred to polyvinylidene fluoride (PVDF) membranes (Millipore, USA). The membranes were incubated with an anti-VIP antibody (Immunostar, USA) overnight, followed by a secondary antibody conjugated to HRP. The signals were visualized using ECL-Plus Western blotting detection reagents (Thermo Fisher Scientific 1,863,096 and 1,863,097). All experiments were repeated three times.

### Quantitative Real‑Time PCR (qRT‑PCR)

qRT-PCR was performed according to our previously reported protocols (Zhang et al. 2022). Briefly, total RNA was extracted using TRIzol reagent (Takara, Japan). A total of 1000 ng of RNA from each sample was converted to cDNA. The cDNA was diluted 1:5 for qRT-PCR and each sample was analyzed in triplicate. Each reaction contained of 0.8 µL primer (Tsingke Biotechnology, Beijing, China), 2 µL cDNA, 10 µL SYBR 2X Master Mix (Vazyme, Nanjing, China) and 7.2 µL DEPC-treated water and amplification was performed after preincubation. The relative expression of the *Vip* gene was calculated using the comparative threshold cycle (2 − ΔΔCt) method (Livak and Schmittgen 2001). The primers used were: *Vip*-F (TGCTGTTCTCTCAGTCGCTG) and *Vip*-R (GCTCCTTCAAACGGCATCCT).

### Immunofluorescence Staining and Fluorescence Imaging

Immunofluorescence staining was performed as described in our previous work (Zhang et al. 2021b). In short, after anesthetization, eyeballs were collected, the cornea, iris and lens were removed, then fixed, dehydrated with 30% (w/v) sucrose solution, and cut into 15-µm-thick cryosections. Then, the sections were washed, blocked with blocking solution, and incubated with primary antibodies overnight at 4 °C. Then, the sections were incubated with Alexa Fluor 488- or 546-conjugated secondary antibody for 1 h at room temperature. DAPI (1:4,000; Beyotime, Shanghai, China; C1002; Hangzhou Dianrui Technology Co., LTD) was used to label the nuclei. Anti-VGAT (1:400; Synaptic Systems; 131,013; Hangzhou Dianrui Technology Co., LTD)(Chen et al. 2022), anti-PSD95 (1:250; Sigma‒Aldrich; MAB1596; ; Hangzhou Dianrui Technology Co., LTD)(Chen et al. 2022), anti-synaptophysin (1:500; Abcam, MA, United States; ab14692; Hangzhou Dianrui Technology Co., LTD)(Scaramuzzino et al. 2022), anti-VIP (1:200; Abcam, MA, United States; ab272726; Hangzhou Hulk Technology Co., LTD)(Casalia et al. 2021) and anti-gephyrin (1:250; Synaptic Systems; 147,021; Hangzhou Dianrui Technology Co., LTD)(Chen et al. 2022) primary antibodies were used and donkey anti-rabbit IgG (1:1000; Thermo Fisher Scientific, MA, United States; A-21,206; Hangzhou Dianrui Technology Co., LTD) (Chen et al. 2022) was used as the secondary antibody.

For determination of the VIP-AC soma number, images of the retinal sections were acquired with a VS120 virtual digital slice scanning fluorescence microscope (6 slice system) (VS120, Olympus, Japan, wide-field, 546 nm, 488 nm and 405 nm filter, software for image capture: VS-ASW-S6). For imaging, we adjusted the excitation intensity of the mercury lamp to visualize the minimum outline of cell soma. When taking images for determination the VIP-AC dendrite number and of some retinal sections in which synapses were labeled, a confocal microscope (FV1000; Olympus, Japan, confocal, 405 and 488 nm filter, software for image capture: FV10-ASW 4.0) with a 20× or 100× oil lens was used. The excitation intensity of the laser was kept constant for the same channel. All the images were taken at room temperature. When we used the spot function of Imaris to match the fluorescence spots representing dendrites, we kept the system threshold constant; this threshold was set to the optimal level for distinguishing weak fluorescence spots from strong fluorescence spots.

### Retinal Slice Preparation

To count the cell somas, we cut the whole eyes into 15-µm-thick slices alone the cornea-optic disk axis. The ten slices between every two collected slices were discarded. To count cell dendrites, we collected 15-µm-thick slices containing the optic nerve.

*For electrophysiological recordings*, mice were euthanized, their eyes were removed, and their retinas were isolated as described previously (Zhao et al. 2018; Li et al. 2017). Briefly, after anesthetization, the eyeballs were rapidly enucleated and placed in ice-cold, oxygenated (95% O_2_ and 5% CO_2_) sucrose-based cutting solution (approximately 4 °C) containing (in mM) 124 sucrose, 26 NaHCO_3_, 10 glucose, 3 KCl, 3 sodium pyruvate, 1.25 NaH_2_PO_4_, 0.2 CaCl_2_, and 3.8 MgCl_2_ (pH 7.4) for one minute. Then, the eyeballs were transferred to filter paper on ice, and the corneas and lenses were cut under a microscope. The retinas were transferred together with part of the filter paper to cutting solution for another three minutes. The retinas were carefully isolated and embedded in low-melting point agarose (4% in artificial cerebral spinal fluid [ACSF]) for four minutes. Vertical retinal slices were cut at a thickness of 200 μm on a vibratome (VT 1200 S, Leica) and incubated in a holding chamber where they were completely submerged in ACSF containing (in mM) 125 NaCl, 25 NaHCO_3_, 10 glucose, 2.5 KCl, 2.5 CaCl_2_, 1.25 NaH_2_PO_4_, and 1 MgCl_2_ (pH 7.4), incubated with 95% O_2_ and 5% CO_2_, and maintained at room temperature for at least 30 min before recording.

### Electrophysiological Recordings

Whole-cell current-clamp recordings were performed using a patch amplifier (Heka Elektronik, EPC 10, Germany) with Patchmaster software, as described in detail in our previous studies (Chen et al. 2022; He et al. 2019). Individual retinal slices were continuously perfused with oxygenated ACSF at a rate of 1–2 ml/min at room temperature. Infrared-differential interference contrast (IR-DIC) video microscopy (Olympus, Japan) was used to identify VIP-tdTomato-ACs in retinal slices. All recordings were made on VIP-ACs mainly in the inner nuclear layer (INL). A horizontal pipette puller (P97, Sutter) was used to pull borosilicate glass pipettes (8–10 MΩ), which were filled with potassium-based intracellular fluid containing (in mM) 120 potassium D-gluconate, 10 4-(2-hydroxyethyl) piperazine-1-ethanesulfonic acid (HEPES), 4 ATP-Mg, 0.5 ethylene glycol-bis (b-aminoethyl ether) N, N, N’, N’-tetra acetic acid (EGTA), 0.3 Tris-GTP, and 15 KCl_2_ (adjusted with KOH to pH 7.3, 280–290 mOsm/L). To record spontaneous excitatory postsynaptic currents (sEPSCs) and spontaneous inhibitory postsynaptic currents (sIPSCs), cesium-based intracellular fluid containing (in mM) 100 CsCH_3_ SO_3_, 20 KCl, 10 HEPES, 7 Tris 2-phosphocreatine, 4 Mg-ATP, 0.3 Tris-GTP and 3 QX-314 (pH 7.3, 285–290 mOsm/L) was used. All experiments were performed at room temperature under normal light conditions. After establishing the whole-cell configuration, the fast and slow capacitance as well as the series resistance (Rs) were carefully adjusted. The amount that the slow capacitance was adjusted was considered the cell membrane capacitance (MC). The Rs was normally less than 33 MΩ, and recordings with a change in Rs exceeding 20% were discarded (Veruki and Hartveit 2002). Seals with a resistance higher than 8 GΩ were considered good seals (Wilson et al. 2011). For resting membrane potential (RMP) measurement, the potential at *I = 0* was recorded in current-clamp model. For current-clamp recordings, no bias current was injected. The input resistance (IR) was calculated as *5mV / (I*_*− 75mV*_*-I*_*− 70mV*_*)* in voltage-clamp model. sEPSCs and sIPSCs were recorded at holding potentials of -70 mV and + 10 mV in regular ACSF, respectively. The E/I ratio of each cell was calculated as E/(E + I) (Antoine et al. 2019; Chen et al. 2022). For potassium current recording, cells were held at − 65 mV, and the voltage was increased from − 65 to 75 mV in 10 mV steps. Among the three subtypes of VIP-ACs (VIP1-ACs, VIP2-ACs and VIP3-ACs) (Perez de Sevilla Muller et al. 2019), VIP1-ACs have gap junctions and likely contribute to the currents measured in voltage-clamp model (Park et al. 2015). Cells were selected as described in previous studies: the main considerations when selecting cells were their location, morphology, glossy and fluorescence layers (Perez de Sevilla Muller et al. 2019). Electrophysiological recordings were digitized at 50 kHz and filtered at 2.0 kHz. MiniAnalysis (Synaptosoft) was used to analyze individual events. Single events were strictly selected according to previously reported standards, namely, a stable baseline, a sharp rising phase, an exponential decay and a single peak (Zhou et al. 2009; Shen et al. 2016). At each set developmental time point, 10–15 cells from three mice were recorded.

### Data Analysis

This is a descriptive study and according to previous similar researches, 2–5 mice is adequate(Liang et al. 2018). In each experiment, we used three to six mice. Three to five cells were recorded in each retina. The number of somas was quantified in 20× sections using ImageJ (Fiji) software. We used the spot function of Imaris 9.0.1 for masking and to determine the spine number as previously described (Zhang et al. 2021b). Electrophysiological recordings were analyzed using MiniAnalysis (Synaptosoft, Leonia, NJ, USA) and Igor 4.0 (WaveMetrics, Lake Oswego, OR, USA). One-way analysis of variance (ANOVA) with Dunnett’s multiple comparisons test (normally distributed) or the Kruskal‒Wallis test with Dunn’s multiple comparisons test (variance was not heterogeneous) was used as appropriate. A value of *p* < 0.05 was considered to indicate significance. “*” refers to comparisons of the initial time point with other time points. “#” refers to comparisons throughout the whole development period. The data were statistically analyzed with GraphPad Prism 8, and the means ± standard deviation (S.D.s) are presented. The picture acquiring, cell recording and data analyzing were performed by different people. To minimize subjective bias, the previous handling people was asked to label the sample in a different sequence according to an appointed rule and the rule will not be open until all the data analyzing were finished. The figure panels were organized into multipart figures with Adobe Illustrator CC 2018.

## Results

### Labeling of Retinal VIP-ACs

**Fig. 1:**
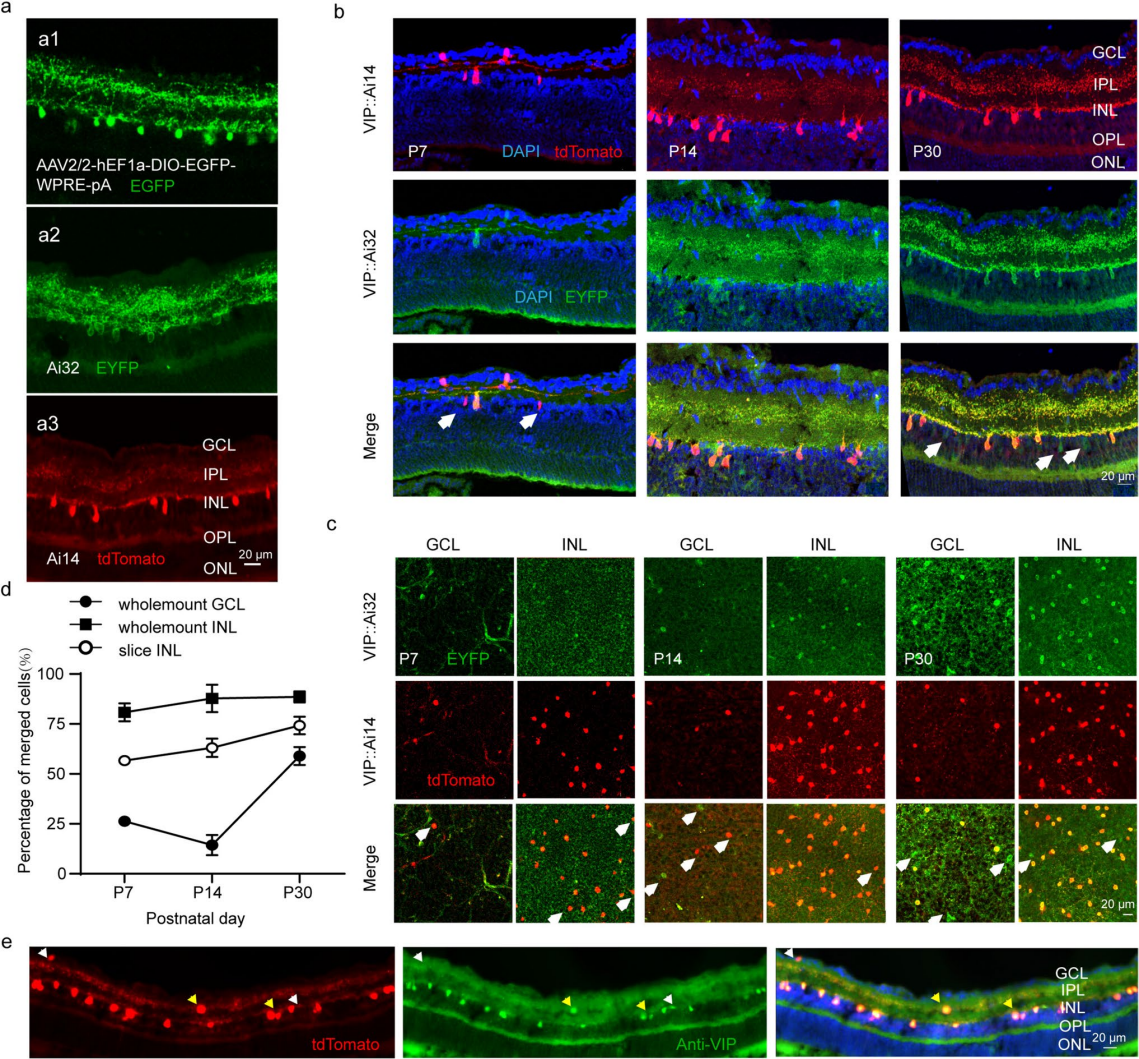
Distribution of VIP-AC in early retinal development. **a** VIP-ACs labeled by Cre-dependent EGFP virus injection into the vitreous cavity of VIP-IRES-Cre mice (a1), mating of VIP-IRES-Cre mice with Ai32 mice (a2), and mating of VIP-IRES-Cre mice with Ai14 mice (a3). **b** EYFP and tdTomato show that the VIP-ACs are colabeled by Ai14 and Ai32 in retinal sections. **c** EYFP and tdTomato show that the VIP-ACs are colabeled by Ai14 and Ai32 in retinal whole mounts. **d** The proportion of VIP-ACs jointly labeled by Ai14 and Ai32 at each developmental time point was large enough. **e** Immunostaining shows VIP-ACs in the INL and GCL (red: tdTomato; green: rabbit anti-VIP; blue: DAPI). The yellow arrows indicate cells with good colabeling, while the white arrows show cells labeled with a single marker. Scale bars = 20 μm; n = 4–6 mice; the data are presented as the mean ± S.D.

We labeled retinal VIP-ACs by three methods. First, VIP-IRES-Cre mice were injected with a Cre-dependent enhanced green fluorescent protein (EGFP)-expressing virus into the vitreous cavity so that VIP-ACs expressed EGFP and exhibited green fluorescence (Fig. 1a1). Second, VIP-IRES-Cre mice were bred with Ai32 mice so that VIP-ACs expressed enhanced yellow fluorescent protein (EYFP) and exhibited yellow‒green fluorescence (Fig. 1a2). Third, VIP-IRES-Cre mice were mated with Ai14 mice so that VIP-ACs expressed tdTomato and exhibited red fluorescence (Fig. 1a3). We found that the cell bodies of VIP-ACs were filled with red fluorescence in VIP::Ai14 mice, whereas yellow‒green fluorescence was observed only on the cell membrane and dendrites of VIP-ACs in VIP::Ai32 mice. To further observe the ability of Ai14 and Ai32 to co-label VIP-ACs, VIP::Ai32 mice were mated with Ai14 mice. Good colabeling of VIP-ACs was observed in both retinal sections (Fig. 1b) and whole mounts (Fig. 1c) at different developmental time points. According to statistical analysis (Fig. 1d), the proportion of colabeled VIP-ACs in the INL at each developmental time point was very high (sections P7 [75.93 ± 14.33]%; P14 [66.91 ± 15.68]%; P30 [85.84 ± 7.33]%; whole mounts: P7 [75.93 ± 14.33]%; P14 [66.91 ± 15.68]%; P30 [85.84 ± 7.33]%). However, the percentage of colabeled VIP-ACs in the GCL was low (P7 [31.55 ± 2.05]%; P14 [21.15 ± 6.29]%; P30 [60.04 ± 11.85] %), especially before eye opening. This may have been because the YFP fluorescence intensity of Ai32 is weak and because Ai32 YFP fluorescence is not easily observed in thick retinal whole mounts. However, in adulthood, the proportion of colabeled VIP-ACs reached more than 60%. Therefore, the overall conclusion is that the colabeling of VIP-ACs in VIP::Ai32 mice and VIP::Ai14 mice is strong and comparable. Moreover, VIP and the transgenic marker were strongly colocalized in VIP::Ai14 mouse retinas, the percentage of colabeling cells was (94.00 ± 0.21)% (Fig. 1e), with approximately 6% cells were only labeled by anti-VIP or tdTomato. However, to ensure the accuracy of the experiment, we selected a single mouse strain, specifically the VIP::Ai14 strain, as VIP-AC somas could be more easily visualized in these mice. VIP::Ai32 mice are more likely to be useful for the study of synapses because of the weak fluorescence intensity of YFP in VIP-ACs.

### Changes in the Development of VIP-AC Somas and Total VIP Expression

**Fig. 2:**
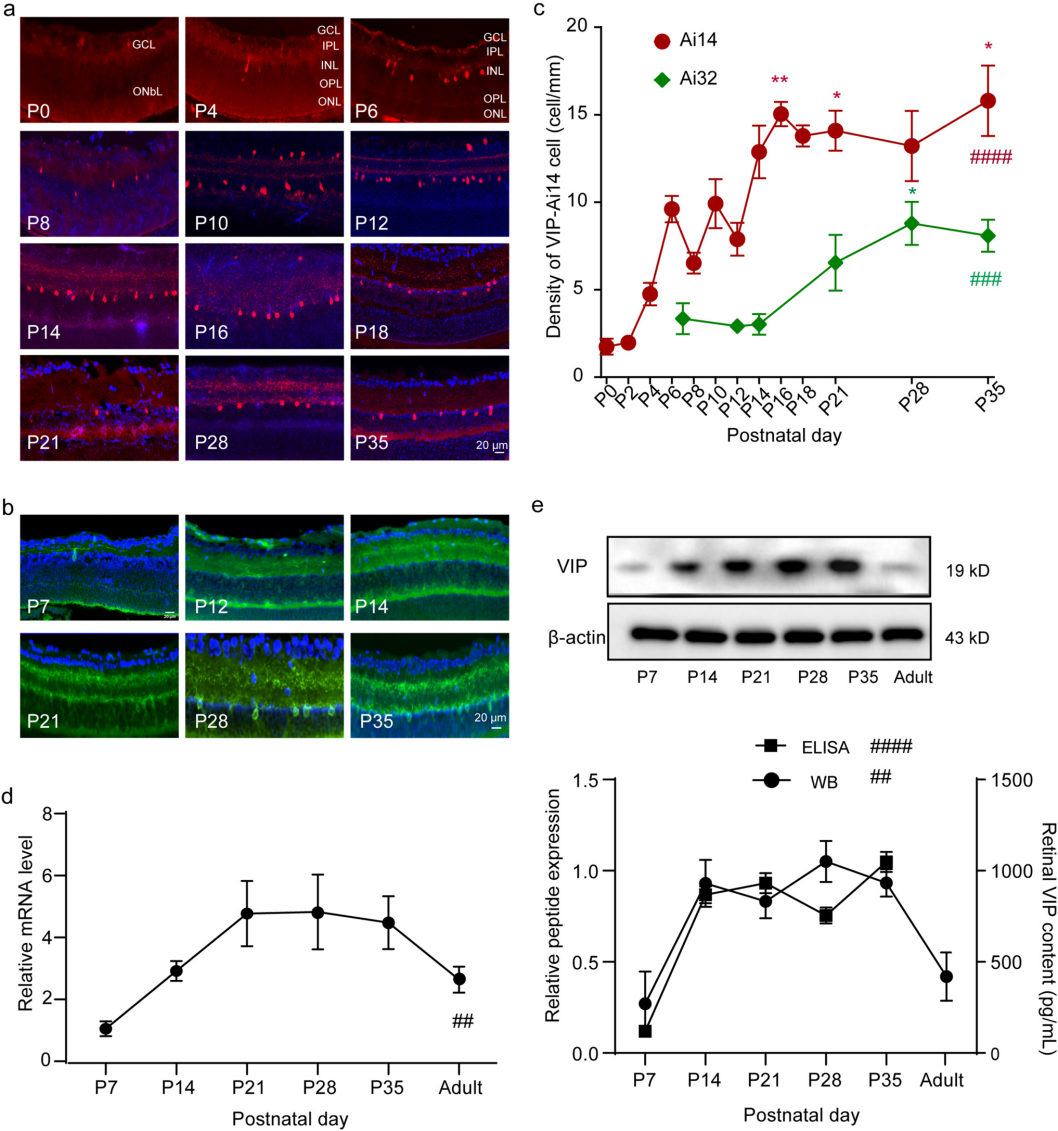
VIP-AC somas and total retinal VIP expression changed greatly during development. VIP-tdTomato-ACs (**a**) and VIP-YFP-ACs (**b**) were used to assess VIP-AC development and analysis (**c**). n = 3–6 mice. Total retinal VIP content determined by qPCR (**d**), WB and ELISA (**e**). n = 3–4 mice. Scale bars = 20 μm in A and B. All data are presented as the mean ± S.D. *p < 0.05; ** and ##p < 0.01; ***and ###p < 0.001; ####p < 0.0001. One-way ANOVA test and the Kruskal‒Wallis test

Both VIP-tdTomato and VIP-YFP mice have been widely used in research (Akrouh and Kerschensteiner 2015; Park et al. 2015; Brecha et al. 2014; Perez de Sevilla Muller et al. 2019). In VIP-tdTomato mice, significant changes in the number and location of VIP-ACs were observed during development (Fig. 2a and c, Kruskal‒Wallis test, Kruskal-Wallis statistic = 70.69, *p* < 0.0001). VIP-ACs were first observed in the innermost portion of the outer neuroblastic layer (ONbL) from P0. At P4, the soma were long and thin and observed on both sides of the inner plexiform layer (IPL). The cell number reached the first peak at P6 (9.61 ± 1.31 cells/mm) and was slightly decreased at P8 (6.52 ± 1.98 cells/mm); then, it peaked again at P10 (9.92 ± 2.81 cells/mm) and reached an almost stable level at P14 (12.88 ± 3.01 cells/mm). In VIP-YFP mice, VIP-AC somas exhibited significant changes during development (Fig. 2b and c, Kruskal‒Wallis test, Kruskal-Wallis statistic = 21.33, *p* = 0.0007). We first observed VIP-ACs at P7 (3.35 ± 1.78 cells/mm), later than in VIP-tdTomato mice. At this time, somas were already present on both sides of the IPL. The developmental trend was the same for the two mouse lines, but the density of VIP-YFP-ACs was nearly half that of VIP-tdTomato-ACs (Fig. 2c). Moreover, the density did not peak at P14 (3.02 ± 1.33 cells/mm); instead, it peaked at P28 (8.79 ± 3.03 cells/mm). Similarly, and interestingly, most labeled VIP-ACs were located in the inner nuclear layer (INL) in retinal sections. Total retinal VIP mRNA and protein expression changed significantly during development, as determined by qPCR (Kruskal‒Wallis test, Kruskal-Wallis statistic = 18.7, *p* = 0.0022, Fig. 2d), WB (one-way ANOVA, F = 5.281, *p* = 0.0023) and ELISA (one-way ANOVA, F = 48.13, *p* < 0.0001, Fig. 2e). It increased from P7 (119.30 ± 39.03 pg/mL) and reached a stable level at P14 (868.70 ± 92.92 pg/mL), showing a slight decrease at P28.

### Developmental Changes in VIP-AC Dendrites

**Fig. 3:**
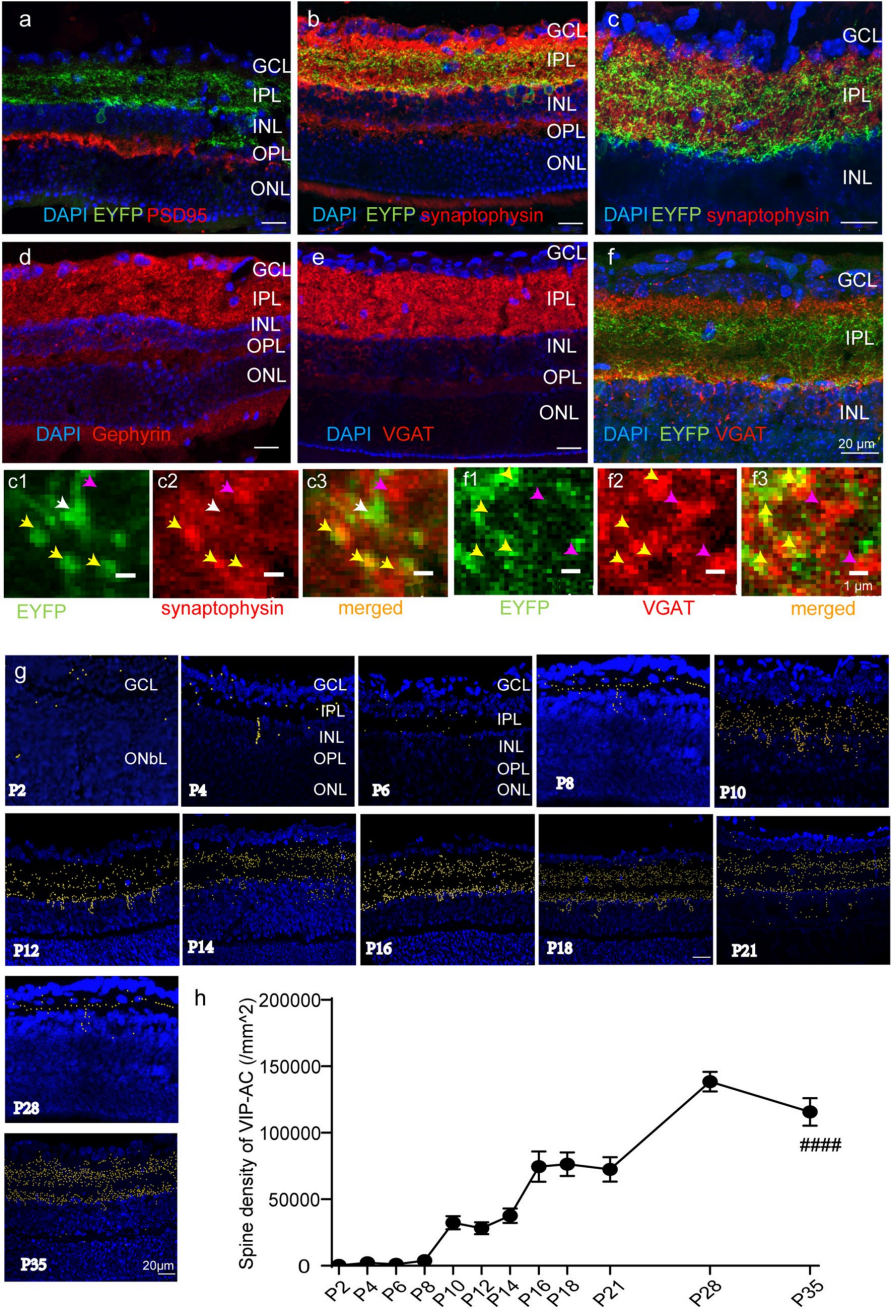
Developmental changes in VIP-AC spines. Immunostaining shows retinal synapses labeled by PSD95 (**a**), synaptophysin (**b** and **c**), Gephyrin (**d**) and VGAT (**e** and **f**). Enlarged pictures of synaptophysin (**c**1–**c**3) and VGAT (**f**1–**f**3) labeling. Spines were matched by spot of Imaris (**g**) and analyzed (**h**). The scale bars are 20 μm in **a**–**f** and **g** and 1 μm in **c**1–**c**3 and **f**1–**f**3. (**g**) Imaris spot-fitting maps of VIP-AC synapses at different time points. Scale bars = 20 μm. **h** Statistical analysis of the number of VIP-AC synapses. n = 3–6 mice. All data are presented as the mean ± S.D. ####p < 0.0001. One-way ANOVA

The IPL and outer plexiform layer (OPL) are the two dendritic layers in the retina. PSD95-labeled excitatory postsynaptic structures were mainly found in the OPL (Fig. 3a), whereas synaptophysin-labeled excitatory and inhibitory presynaptic structures were observed in both the IPL and OPL (Fig. 3b). In the IPL, synaptophysin and EYFP dendrites were almost colocalized (Fig. 3c). Gephyrin-labeled inhibitory postsynaptic structures were found mainly in the IPL (Fig. 3d), while VGAT-labeled inhibitory presynaptic structures were found in both the OPL and IPL (Fig. 3e and f). From the enlarged pictures, we can see that approximately 90% YFP-positive spines expressed high level of synaptophysin or VGAT, whereas approximately 4% YFP-positive spines did not express any synaptophysin or VGAT, and approximately 6% YFP-positive spines expressed low level of synaptophysin (Fig. 3c1–c3, f1–f3). This indicated that YFP labeled the dendritic spines of VIP-YFP-ACs, as reported previously (Zhang et al. 2021b). With the spot function of Imaris, we evaluated the spine development of VIP-ACs (Fig. 3g and h). At P2, the IPL was thin, and the number of VIP-AC spines was very small (285.4 ± 70.3/mm^2^). Afterward, the IPL became wider, and the spine density continued to increase. At P28, the spine density peaked (138,351.3 ± 14,673.0/mm^2^), followed by a slight decrease at P35.

### Developmental Changes in the Passive Membrane Properties of VIP-ACs

**Fig. 4:**
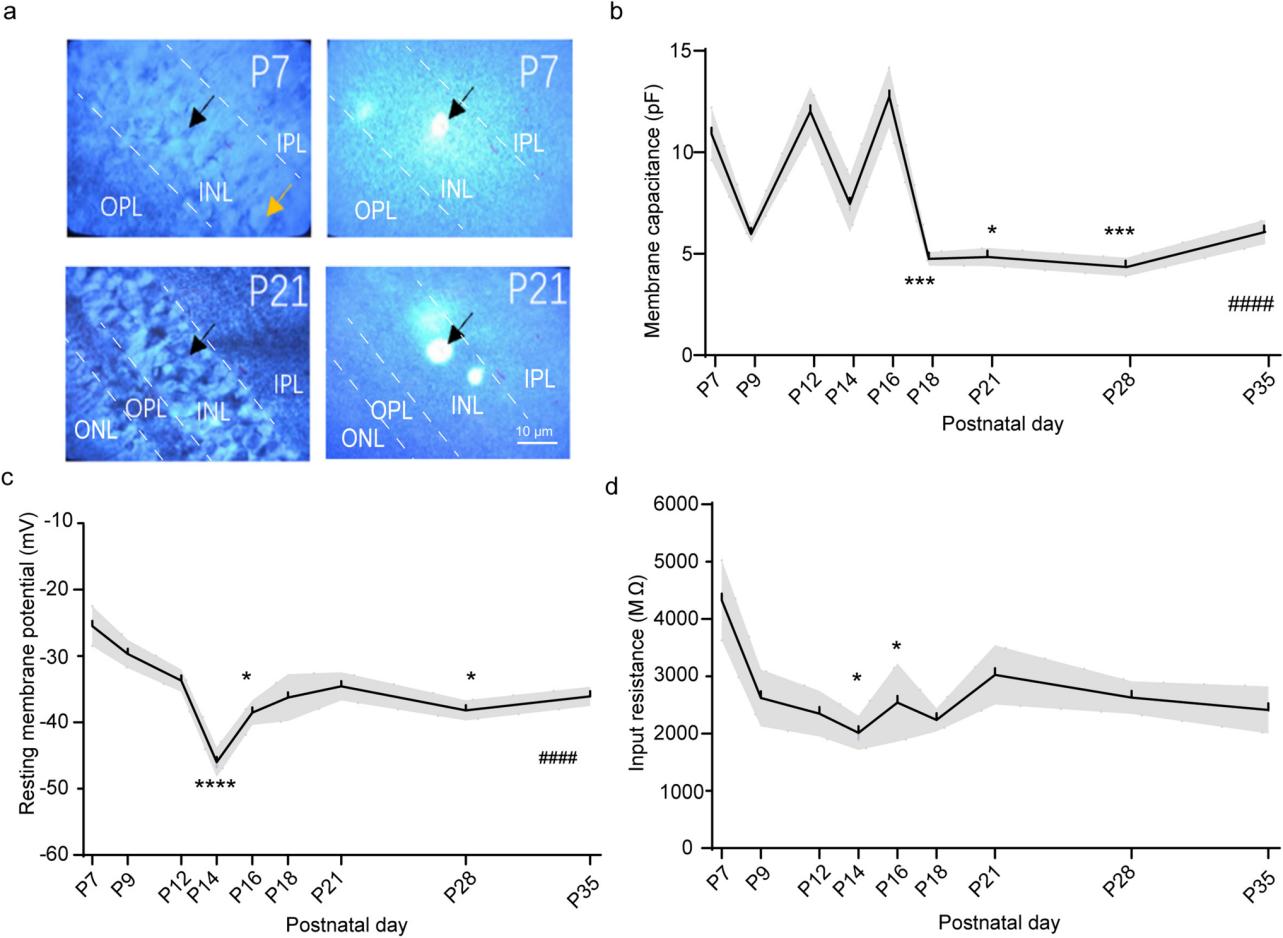
Developmental changes in the passive membrane properties of VIP-ACs. **a** Under IR-DIC video microscopy, cells were observed without (left) or with (right) fluorescence. Cell MC (**b**), RMP (**c**) and IR (**d**) show different change trends. n = 3–5 mice and only the right eyes were used for recording. 3–5 cells were recorded in each retina. All data are presented as the mean ± S.D. *p < 0.05; **p < 0.01; ***p < 0.001; **** and ####p < 0.0001. Kruskal‒Wallis test

In addition to cell and spine density, the function of VIP-ACs also changed during development. Under IR-DIC video microscopy, we observed that at P7, the cell somas had diverse diameters, and fluorescent cells were clearly seen; however, at P21, the cell diameter became more uniform (Fig. 4a). We measured the cell MC in current-clamp model and found that it changed significantly during development (Fig. 4b, Kruskal‒Wallis test, Kruskal-Wallis statistic = 56.42, *p* < 0.0001). It was high at P7, P12 and P16 (10.65 ± 5.19 pF, 11.75 ± 4.33 pF, and 12.47 ± 6.28 pF, respectively), low at P9 and P14 (5.72 ± 1.65 pF and 7.20 ± 5.88 pF, respectively) and reached the adult level at P18 (4.50 ± 1.79 pF). The RMP significantly changed during development (Kruskal‒Wallis test, Kruskal-Wallis statistic = 40.10, *p* < 0.0001). The RMP decreased from P7 to P14 during VIP-AC development and reached the lowest value at P14 (− 46.05 ± 8.02 mV). Then, it increased again, and at approximately P18, it reached a stable level (− 36.27 ± 13.68 mV) (Fig. 4c). The IR did not change significantly during development (one-way ANOVA, F = 1.605, *p* = 0.1361). The change trend of the IR was similar to that of RMP, first decreasing and then increasing. However, it reached the lowest point at P16 (2,643.00 ± 1,410.00 MΩ), which was later than when the RMP reached the lowest point. It reached a stable level at approximately P21 (3,929.00 ± 2,544.00 MΩ) (Fig. 4d).

### Developmental Changes in VIP-AC Potassium Currents

**Fig. 5:**
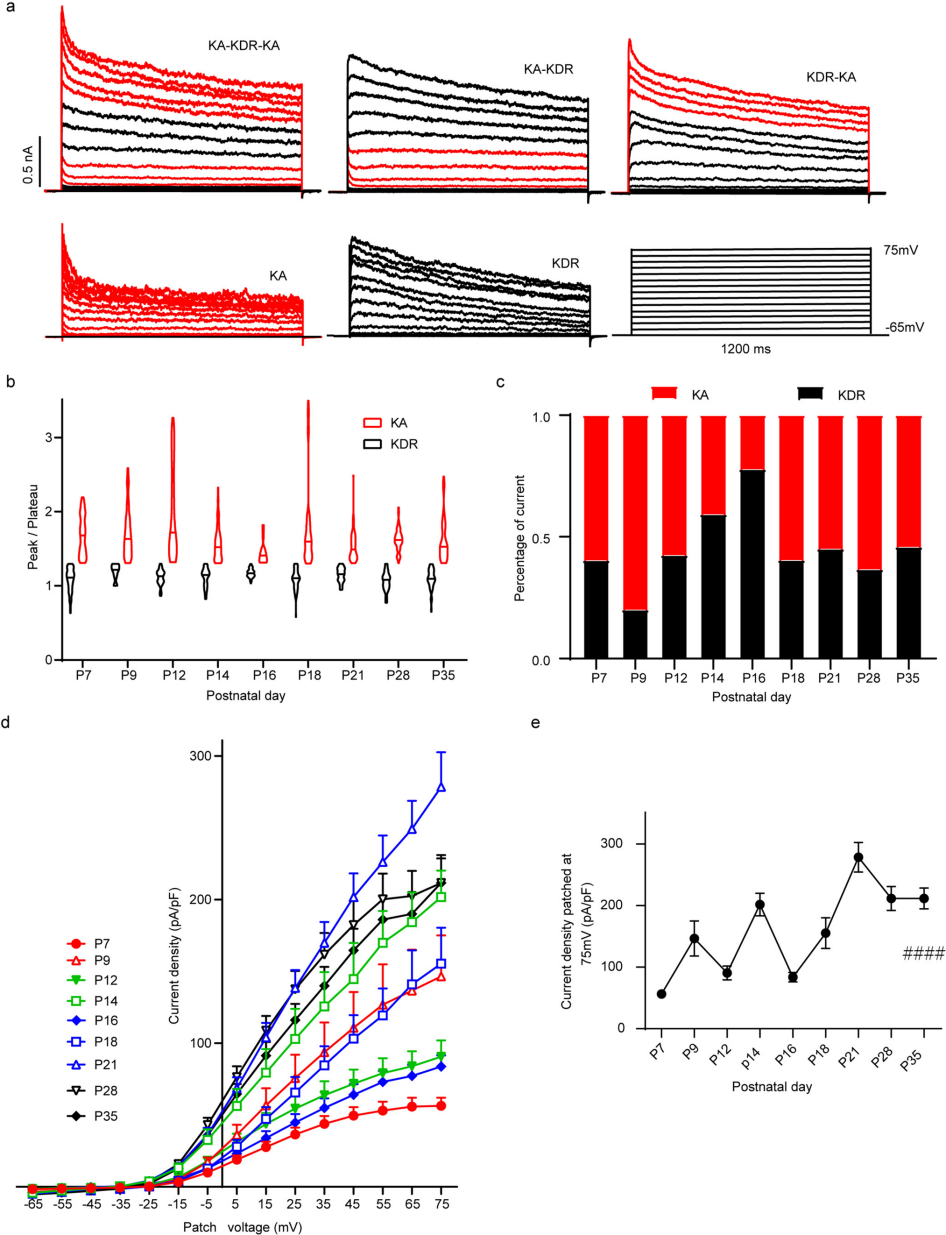
Developmental changes in VIP-AC K^+^ currents. **a** Five subtypes of total K^+^ currents. The different K^+^ currents distinguished by peak/plateau ratios and the developmental characteristics of K_A_ and K_DR_ (**b**) and their percentages (**c**). **d** Developmental changes in the I–V curves of *I − K*_*total*_. **e** Developmental changes in the current density of *I − K*_*total*_. n = 3 − 5 mice and only the right eyes were used for recording. 3–5 cells were recorded in each retina. All data are presented as the mean ± S.D. ####*p* < 0.0001. One-way ANOVA test and the Kruskal‒Wallis test

In voltage-clamp mode, we recorded the total potassium (K^+^) current (*I − K*_*total*_). After holding at a voltage of − 65 mV, we recorded 15 current sweeps as the voltage was increased from − 65 mV to 75 mV in 10 mV steps. We found that in VIP-ACs, there were five types of *I − K*_*total*_, each consisting of two main *I − K* components, *I − K*_*transient*_ (rapidly inactivating K^+^ current, *K*_*A*_) and *I − K*_*sustained*_ (voltage-dependent K^+^ current, *K*_*DR*_) (Robinson and Wang 1998; Guenther et al. 1999; Schnoebel et al. 2005). We named the five types of *I − K*_*total*_ as *K*_*A*_* − K*_*DR*_* − K*_*A*_, *K*_*A*_* − K*_*DR*_, *K*_*DR*_* − K*_*A*_, *K*_*A*_ and *K*_*DR*_ (Fig. 5a). We calculated the I − K peak/plateau index and found that the index for *K*_*A*_ was usually higher than that for *K*_*DR*_. Generally, the index for *K*_*A*_ was larger than 1.3. Analysis of the overall index for each sweep of *I − K*_*total*_ showed that from P7 to P16, the mean index became slightly higher and then lower, and the index became less varied. Then, the mean index and the variable of the index increased at P18 and reached a stable level (1.5 ± 0.015, range: 0.6–3.5). We analyzed the *K*_*A*_ and *K*_*DR*_ components separately by using an index cutoff of 1.3 and found that the percentage of the *K*_*DR*_ component remained nearly the same from P7 to P35; however, the percentage of the *K*_*A*_ component first slightly increased and then decreased at P16, thereafter reaching a stable level (Fig. 5b). Another obvious change during development was that the percentage of the *K*_*DR*_ component in *I − K*_*total*_ peaked at P16 (77.8%) and reached the lowest point at P9 (20%) (Fig. 5c). The I–V curve of *I − K*_*total*_ showed that all of these currents had outward rectifier characteristics (Fig. 5d). The current density significantly increased during development (Kruskal‒Wallis test, Kruskal–Wallis statistic = 72.54, *p* < 0.0001), with three peaks at P9, P14 and P21 (146.50 ± 90.18 pA/pF, 201.70 ± 48.64 pA/pF, 278.50 ± 90.16 pA/pF, respectively) (Fig. 5e).

### Developmental Changes in Spontaneous EPSCs and IPSCs in VIP-ACs

**Fig. 6:**
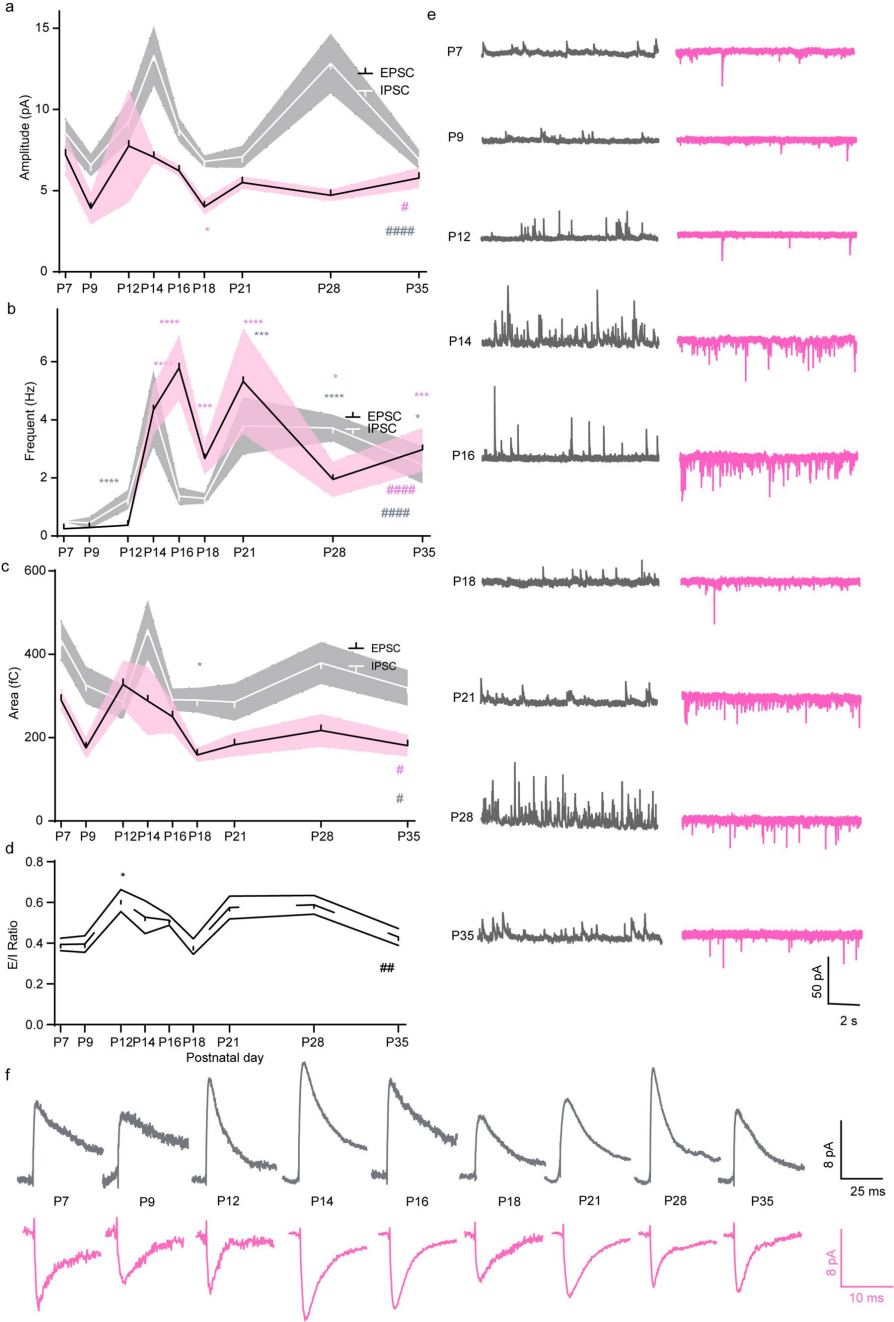
Developmental changes of sEPSCs and sIPSCs in VIP-ACs. The amplitude (**a**), frequency (**b**) and charge transfer (**c**) of sEPSCs and sIPSCs change specifically during development. The E/I ratio was steady (**d**). **e** and **f** are representative sEPSC and sIPSC traces. n = 3 − 5 mice and only the right eyes were used for recording. 3  − 5 cells were recorded in each retina. All data are presented as the mean ± S.D. * and #*p *< 0.05; ** and ##*p *< 0.01; ***and ###*p *< 0.001; **** and ####*p *< 0.0001. One-way ANOVA test and Kruskal‒Wallis test

We found that the sEPSC amplitude changed significantly but not dramatically throughout development (one-way ANOVA, F = 2.191, *p* = 0.036), ranging from 4 pA to 8 pA. Specifically, the sEPSC amplitude peaked twice at P7 (7.20 ± 5.08 pA) and P12 (7.75 ± 6.93 pA) and reached the lowest point twice at P9 (3.91 ± 1.38 pA) and P18 (4.01 ± 2.19 pA). At P21 (5.49 ± 1.21 pA), the amplitude reached a stable level (Fig. 6a). However, the frequency was very low from P7 to P9 (0.29 ± 0.06 Hz at P7), and dramatically increased beginning on P12 and reached its first peak at P16 (5.78 ± 3.13 Hz). Surprisingly, at P18, the frequency decreased again (2.67 ± 2.48 Hz). Then, it increased again, reached a second peak at P21 (5.32 ± 5.73 Hz) and then slightly decreased to a stable level (2.97 ± 2.13 Hz at P35). Overall, the sEPSC frequency significantly changed during development (Kruskal‒Wallis test, Kruskal-Wallis statistic = 49.4, *p* < 0.0001) (Fig. 6b). While the variation in the amplitude was large at P9 to P12, the variation in the frequency was small. The opposite change was observed at later stages, except for at P18, when the variability in both the frequency and amplitude was small. However, the charge transfer significantly changed during development (one-way ANOVA, F = 2.043, *p* = 0.0471), peaked at P12 (327.60 ± 171.7 fC), and slowly decreased until P18 (158.30 ± 71.2 fC), when it reached the adult level. The variability in the charge transfer was large only at P12 and at P14, and it remained stable from P18 (Fig. 6c). In summary, dramatic changes in sEPSCs occurred beginning at P12, with the sEPSC amplitude peaking at P14, surprisingly decreasing at P18 and reaching a stable level at P21.

The sIPSC amplitude was larger than the sEPSC amplitude and significantly changed during development (Kruskal‒Wallis test, Kruskal-Wallis statistic = 36.83, *p* < 0.0001), ranging from 6 pA to 15 pA. The sIPSC amplitude peaked twice at P14 (13.31 ± 7.51 pA) and P28 (12.83 ± 8.39 pA), later than the sEPSC amplitude. Additionally, the sIPSC amplitude reached the lowest level at P9 (6.57 ± 2.57 pA) and P18 (6.80 ± 1.87 pA) (Fig. 6a). The sIPSC frequency also changed significantly during development (Kruskal-Wallis test, Kruskal-Wallis statistic = 68.71, *p* < 0.0001), peaking twice at P14 (4.38 ± 5.24 Hz) and P21 (3.79 ± 3.39 Hz). The first peak appeared earlier than the first sEPSC frequency peak (Fig. 6b). Charge transfer also significantly changed during development (Kruskal‒Wallis test, Kruskal-Wallis statistic = 17.69, *p* = 0.0237); charge transfer peaked at P14 (459.00 ± 302.9 fC), which was later than the sEPSC charge transfer peak, and quickly decreased to the adult level at P16 (291.10 ± 112.8 fC) (Fig. 6c), leading to an increase in the E/I ratio from P7 to P12 and a decrease from P12 to P18 (one-way ANOVA, F = 3.393, *p* = 0.0045) (Fig. 6d). The variability of all the parameters was similar. Figure 6e and f show representative traces.

## Discussion

**Fig. 7:**
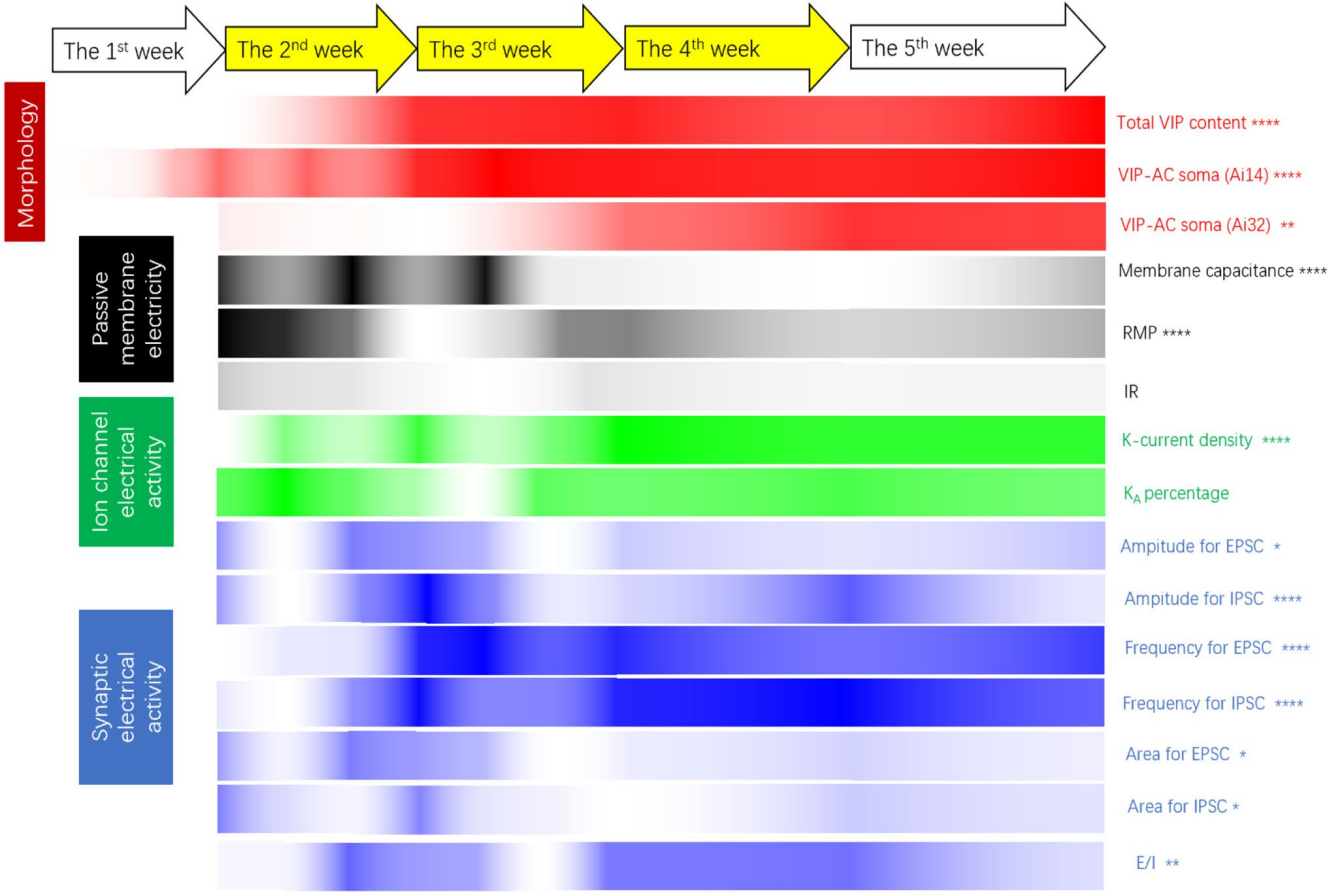
Schematic diagram showing the details of the developmental changes in all the parameters. After birth, all the measured parameters changed dramatically. For each bar, a lighter colour indicates a smaller value; thus, there were many peaks and valleys. Overall, the second, third and fourth weeks after birth were important periods of VIP-AC development

The current work systematically describes the developmental changes in the structure and function of retinal VIP-ACs during the peri-eye-opening period for the first time (Fig. 7). As previously reported, the number and electrophysiological properties of VIP-ACs peaked at approximately P14. However, the spine density continued to increase. After P14, the cell density remained stable, whereas VIP-AC currents showed more diverse changes. This indicated that the developmental trends of VIP-AC structure and function were not parallel and that functional development was more complex and occurred over a longer time period.

The VIP::Ai32 and VIP::Ai14 mouse lines are two widely used mouse lines for analyzing the function and morphology of retinal VIP-ACs (Akrouh and Kerschensteiner 2015; Park et al. 2015; Perez de Sevilla Muller et al. 2019; Brecha et al. 2014). There is a nearly complete overlap between the VIP-tdTomato and VIP-ChR2-EYFP ACs in the adult mice. However, during early develop stages, the discrepancy in the density of labeled VIP-ACs in the two VIP lines (Fig. 2c) found in the current manuscript may be due to technical issues, such as faster bleaching of the membrane-bound EYFP and thus detect ability, as well as delayed expression of ChR2-EYFP compared to tdTomato, or EYFP has a worse expression as it’s a second cassette after ChRh in the Rosa26Ai32 construct. So, VIP::Ai14 mouse line is better for soma analysis and electrophysiological recording, whereas VIP::Ai32 mouse line is more suitable for synapse analysis. Moreover, we observed that there were very few VIP-ACs in the GCL in retinal sections from VIP::Ai32 and VIP::Ai14 mice. In virus-injected mice, VIP-ACs in the GCL accounted for only approximately 2% of all VIP-ACs. Therefore, in our current study, we mainly focused on cells in the INL.

### The VIP-AC Soma Density Plateaus During Development, While Developmental Changes in Spines Continue

Previous studies have reported that the cell density of VIP-ACs as well as other ACs usually peaks at approximately P15 (the point of eye opening) (Casini et al. 1994; Bagnoli et al. 2003). According to analysis of a fluorescence reporter mouse line and measurement of total retinal VIP content by ELISA and WB, the VIP-AC number and total retinal VIP content peaked at P14 (the point of eye opening in mice). However, we did not observe a subsequent dramatic decrease in the VIP-AC number or total retinal VIP content; instead, we observed that these parameters plateaued at a high level. In previous studies, observations were made at a few time points before adulthood. In mice, adulthood usually refers to ages greater than six weeks, and in our experiment, we indeed observed a decrease in total retinal VIP levels by WB. The increasing trend in density for VIP-ACs was not the same as that observed for proopiomelanocortin (POMC)-ACs (Zhang et al. 2021b). The POMC-AC density peaked slightly earlier than the VIP-AC density, i.e., at P12. After P14, the density did not plateau. This indicated that different types of ACs participate in retinal development at diverse time points. The VIP-AC density in VIP::Ai32 mice was very low, and details about the VIP-AC density could not be determined; thereafter, the VIP::Ai32 mouse line was not a good tool for studying soma and cell density during development.

However, the VIP::Ai32 mouse line was more appropriate for spine analysis because the spines were clearer and denser. Through costaining with different synaptic markers, we confirmed that the observed spots were spines (McGuier et al. 2019), consistent with previous reports (Zhu et al. 2020; Huang et al. 2021; Cueva et al. 2002). We found that unlike the total retinal VIP content and the soma density, the VIP-AC spine density changed from P7. This indicated that at P14, although the cell density was dramatically increased, the spine density was not. In other words, the cell number peaked at P14, but the spine density continued to change until P28. This was consistent with the general developmental rule that the cell number increases first, and then dendrite generation occurs (Horsburgh and Sefton 1987).

### The Passive Membrane Properties of VIP-ACs Indicate Cell Differentiation, and K^+^ Currents Play a Role in These Properties

Changes in cell dendrites and synapses are always accompanied by changes in the cell membrane and ion channels and thus electrophysiological properties, and the electrophysiological properties of VIP-ACs have never been reported. Generally, the number of ACs increases during the embryonic period, and their neurotransmitters are important for the development of RGCs, especially for the connection of axon terminals in the brain (Bagnoli et al. 2003). The IR and RMP are two indicators for measuring the maturity of a cell (Spigelman et al. 1992). When a cell becomes more mature, the levels of ion channels increase, the IR decreases, and the RMP becomes more hyperpolarized. According to our results, the RMP showed an earlier peak at P14. Afterward, the VIP-ACs seemed to immature slightly again, which, however, is theoretically unpractical. Therefore, we speculated that at P14, the cells were undergoing cell differentiation (Das et al. 2005). When a cell undergoes differentiation, similar to when it matures, the RMP becomes more negative. Another theoretical explanation is that from P7 to P14, VIP-ACs experienced an excitatory to inhibitory switch in GABA receptors caused by intracellular chloride concentration ([Cl–](i) decreasing(Zhang et al. 2006). Inhibitory GABA receptors contribute to the negative RMP. However, VIP-ACs were very small, with a diameter of 5–10 μm and a total capacitance of approximately 5 pF, especially after P18. It is difficult to measure the membrane potential of very small cells, but we did observe changes in the RMP, consistent with a previous report (Wilson et al. 2011). We used a strictly controlled protocol and ensured that the seal was good.

The RMP is associated with K^+^ currents. We found five subtypes of outward-rectifying *I − K*_*total*_ in VIP-ACs. Generally, the K^+^ current density increased during development, with two dramatic peaks at P9 and P14; accordingly, the cell MC reached the lowest level at P9 and P14. Apparently, the K^+^ current density and cell MC may directly affect each other. Generally, the density of voltage gated channel, synaptic connectivity and gating charge movements within a channel are all factors that affect the MC(Qian et al. 2014) and they develop inconsistently. Except for potassium channels, the voltage-sensitive sodium channels were also important in retinal ACs (Huba et al. 1992). They present in AC axons at P11 and the density of ring terminals increased markedly from P15 (Witkovsky et al. 2005). This indicated that the first MC peak at P12 is likely due to the function of sodium channels whereas the second MC peak at P 16 contributed by synaptic connectivity. *I − K*_*total*_ can be divided into a transient components, *K*_*A*,_ and a sustained components, *K*_*DR*_ (Guenther et al. 1999). When a cell is undifferentiated, the percentage of *K*_*DR*_ is higher; however, when a cell undergoes differentiation, the percentage of *K*_*A*_ increases (Das et al. 2005; Spigelman et al. 1992). Similarly, according to our results, the percentage of *K*_*A*_ was the highest at P9 and the lowest at P16. This indicated that there was a shift in *K*_*A*_ during the peri-eye-opening period and that cell differentiation occurred. Cell differentiation may have led to the peak in cell density. As previously reported, these aspects of maturation are independent of animal age and most likely proceed according to cell generation and correlate with each other (Liu et al. 2000). Another explanation for the negative RMP is that chloride contributes more to the RMP in immature than in mature neurons (Spigelman et al. 1992). In addition, internal chloride concentrations may contribute to K^+^ conductance. The reason that the peak percentage of *K*_*A*_ was at P9 and not at P14 may have been because the shift from a high to low intracellular chloride concentration occurred before P10 (Zhang et al. 2006). Another important current for achieving a stable RMP is *I*_*K1*_, which is generated by the Kir2.1 (KCNJ2) channel (Giles and Noble 2016). A decrease in Kir2.1 expression led to a hyperpolarized RMP. Therefore, we speculated that during the peri-eye-opening period, changes in K^+^ currents play a more important role than changes in Kir2.1 currents. The developmental trend in the IR was rather smooth, which may have been because the IR reflects total ion channel expression in a cell. The IR can also be influenced by cell integrity. Transection of dendrites can simultaneously increase the IR and decrease the length of dendrites, causing a slight systematic shift toward cellular immaturity (Liu et al. 2000). The procedure we used to obtain retinal slices for recording may have to some extent disrupted cell integrity and caused other alterations obscuring less obvious changes.

### Analysis of Postsynaptic Currents Revealed that the Critical Developmental Period of Excitatory Function is Earlier than that of Inhibitory Function

sEPSCs also changed from P7, with the sEPSC amplitude peaking at P16. Interestingly, during development, the variability in sEPSC frequency increased, while the sEPSC amplitude tended to be more uniform. Usually, frequency is an indicator of neurotransmitter release, and amplitude is an indicator of receptor function (Yang et al. 2018). Therefore, we can speculate that changes in receptor expression occur first, and then neurotransmitter release is modulated. The parameter that changed the most over development was the current kinetics or charge transfer, as indicated by the area analysis. Surprisingly, we noticed that at P18, the frequency of sEPSCs became very low consistent with a decrease in the K^+^ current density. In addition to maintaining the RMP, Kir also regulates the action potential (AP) duration, receptor-dependent inhibition of cellular excitability, and the entry and exit of K^+^ ions through the cell membrane (Isomoto et al. 1997). Therefore, Kir can modulate APs to influence sEPSCs. Paired pre- and postsynaptic recordings confirmed that presynaptic APs evoked EPSCs (Dodson et al. 2003). This may explain our finding that when the K^+^ current density was very low at P18, the frequency of sEPSCs was also very low, although the amplitude was relatively high. At P14 and P16, the amplitude and frequency of sEPSCs were both increased. As mentioned previously, VIP-ACs may undergo differentiation at these stages, and sEPSCs result from presynaptic input from bipolar cells. Glutamate transmission may be important for cell differentiation. The decrease in glutamate transmission at P18 may have represented a mechanism for inhibiting cell differentiation. Others have also found that the frequency of N-methyl-d-aspartate (NMDA)-mediated EPSCs peaks at P15 and then decreases. The NMDA-mediated EPSC amplitude also peaks at P15, while the α-amino-3-hydroxy-5-methyl-4-isoxazole-propionic acid (AMPA)-mediated EPSC amplitude remains high from P15 (Zhang and Warren 2008). This is to some extent consistent with our result; however, observations were not made at P18 in the previous study, and we did not distinguish AMPA- and NMDA-mediated EPSCs. EPSCs can also be induced by the secretion of some neuropeptides. Previous studies have reported that neuropeptide Y (NPY) can decrease the EPSC frequency but not the EPSC amplitude (Rhim et al. 1997). This indicates that a presynaptic mechanism may be involved. For example, NPY suppresses neurotransmitter release at many synapses, primarily through inhibition of Ca^2+^ channels, such as N-type channels (McQuiston and Colmers 1996) or TTX-sensitive sodium channels (Rhim et al. 1997).

The developmental trend of sEPSCs was similar to that of sEPSCs, exhibiting multiple peaks and valleys. However, the time points of the peaks and valleys were different. As a result, the E/I ratio increased before P12 and then decreased. Usually, inhibitory connections develop earlier than excitatory connections due to plasticity (Mapelli et al. 2015). However, by measuring amplitudes and charge transfer, we found that sEPSCs developed earlier than sIPSCs in VIP-ACs. This may have been because GABA neurons form excitatory connections first and then form inhibitory connections later (Zhang et al. 2006). Considering the constant fluctuations in both sEPSCs and sIPSCs until three weeks after birth, we speculated that the critical period of VIP-AC development is much longer than previously reported.

### Limitations and Future Prospects

In adulthood animal models, especially mice, VIP-ACs have been further divided into three main subtypes, according to their characteristic location, morphology, electrophysiological properties and light response. However, our findings revealed that for the total VIP-ACs, the discrepancy between different developmental time points is great but the error bar of the same time point is small. These findings just show that during early developmental stages, it is not proper or is complex to make classification according to morphology or electrophysiological properties. Limited by a useful classifying method, we have not distinguished subtypes of VIP-ACs. We suggest that subtype specific genetic markers may be more useful. Patch-clamp electrophysiology combined with single cell transcription sequencing could be helpful. By analyzing genetic characterization together with morphology and electrophysiological properties in adulthood, we could find genetic markers, and finally confirm these markers to developmental stages is also crucial.

In conclusion, we found that although VIP::Ai14 and VIP::Ai32 mice are both widely used for studying VIP-ACs, they have advantages and disadvantages for different applications. Unlike that of other ACs, the density of VIP-ACs peaked at P14 and did not decrease immediately. However, the electrophysiological properties of VIP-ACs developed specifically from P14 to P18, with more complex developmental changes in passive membrane properties, K^+^ currents, sEPSCs and sIPSCs developments, occurring during the peri-eye-opening period, indicating that VIP-ACs may have an important function in retinal development during a longer critical period. Based on these findings, subtype specific description is needed in the future.

## Data Availability

The datasets generated during and/or analyzed during the current study are not publicly available due to the lack of a public online database but are available from the corresponding author on reasonable request.
